# Supplementary figures and images for: Transcriptome Profiling Reveals the Gene Network Responding to Low Nitrogen Stress in Wheat
Source: Plants (Basel). 2024 Jan 26;13(3):371. doi: 10.3390/plants13030371 (PMC10856819; doi:10.3390/plants13030371)

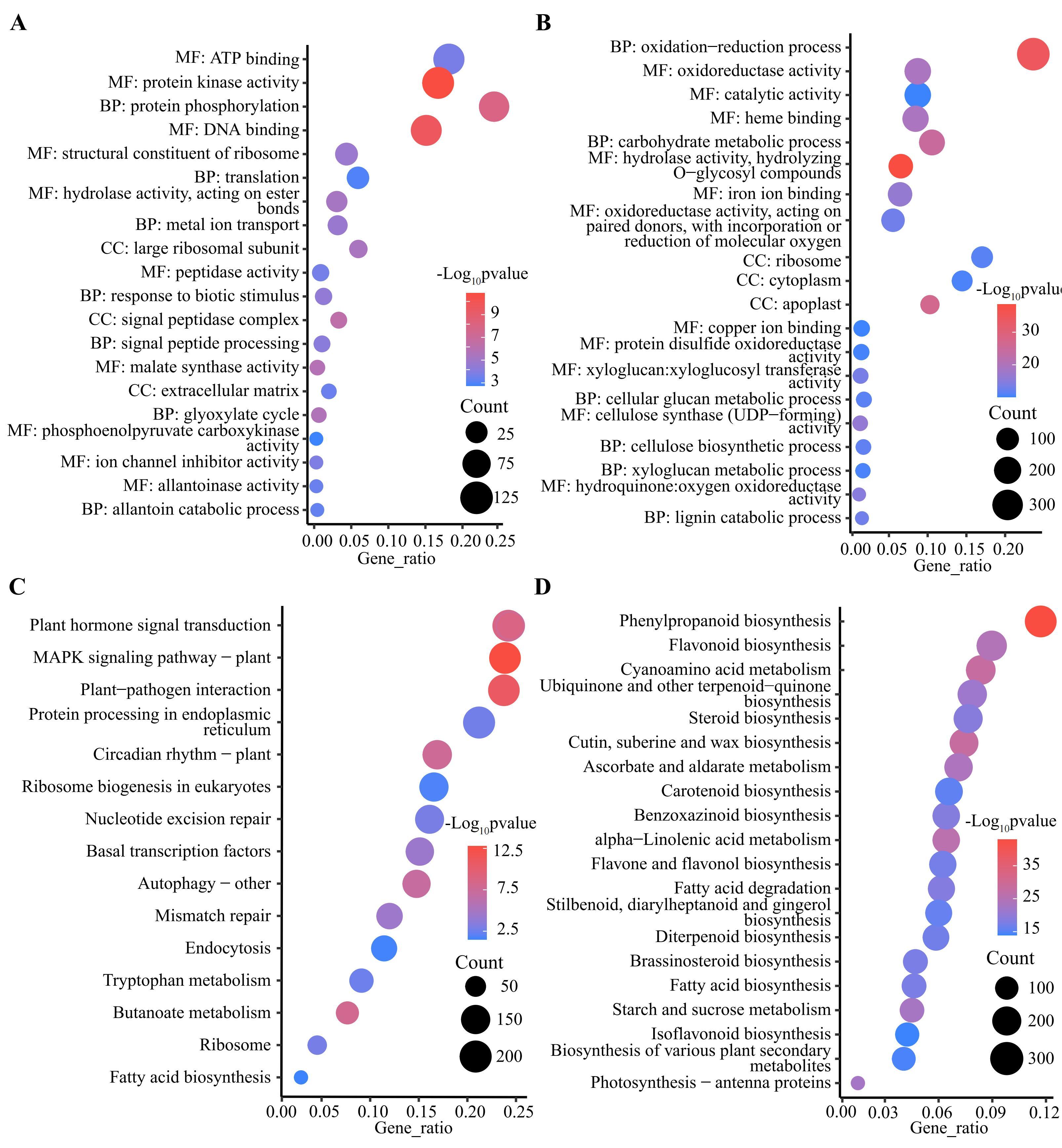

Supplement: Supplementary file 1 [file plants-13-00371-s001.zip › Figure S1 - GO and KEGG enrichment analysis of DEGs in the shoots.jpg]

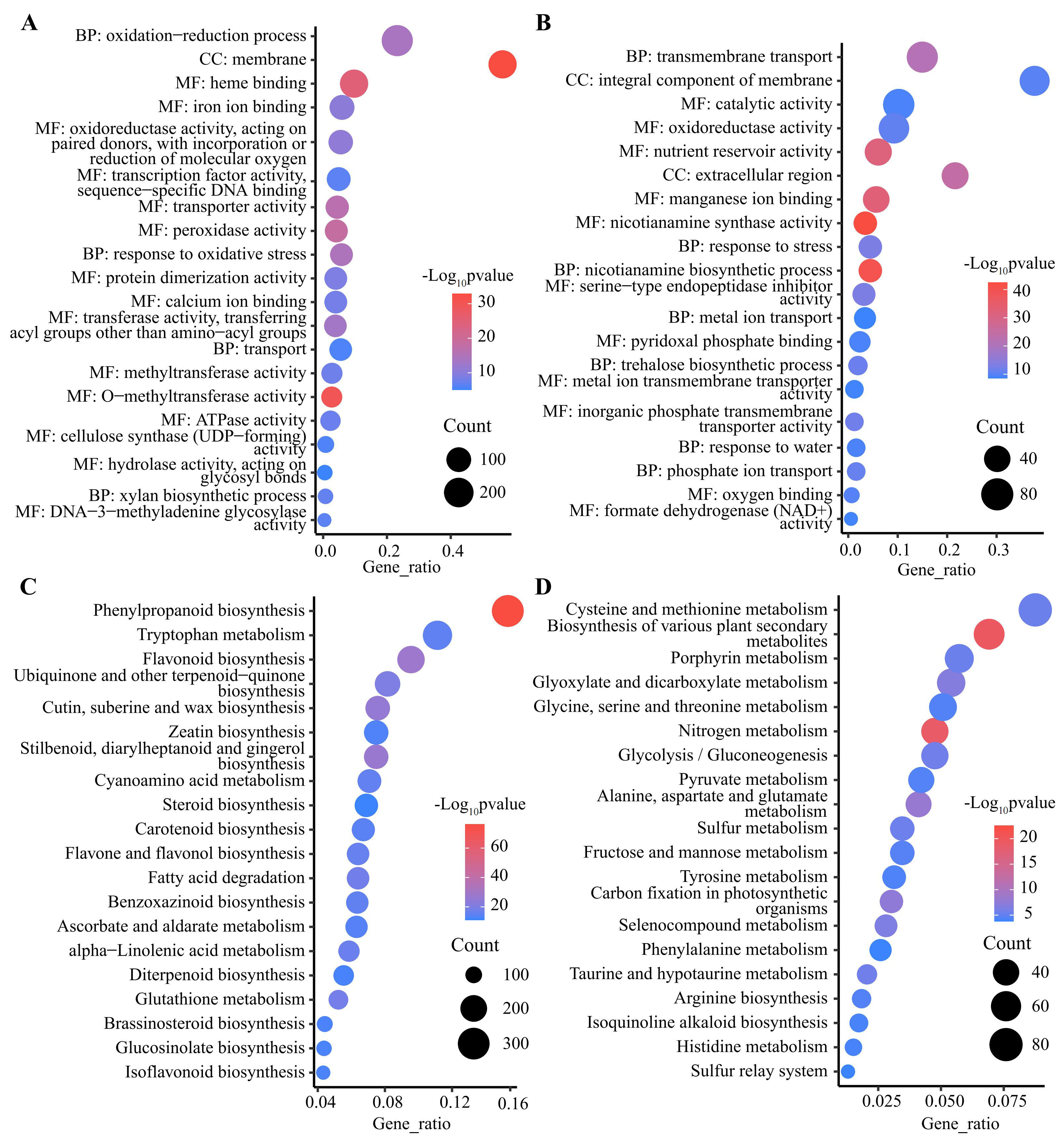

Supplement: Supplementary file 1 [file plants-13-00371-s001.zip › Figure S2 - GO and KEGG enrichment analysis of DEGs in the roots.jpg]
